# Two Different Macaviruses, *ovine herpesvirus-2* and *caprine herpesvirus-2*, Behave Differently in Water Buffaloes than in Cattle or in Their Respective Reservoir Species

**DOI:** 10.1371/journal.pone.0083695

**Published:** 2013-12-27

**Authors:** Anina B. J. Stahel, Rhea Baggenstos, Monika Engels, Martina Friess, Mathias Ackermann

**Affiliations:** 1 Institute of Virology, Vetsuisse Faculty, University of Zurich, Zurich, Switzerland; 2 Department of Farm Animals, Vetsuisse Faculty, University of Zurich, Zurich, Switzerland; University of Liverpool, United Kingdom

## Abstract

The ongoing global spread of “exotic” farm animals, such as water buffaloes, which carry their native sets of viruses, may bear unknown risks for the animals, into whose ecological niches the former are introduced and vice versa. Here, we report on the occurrence of malignant catarrhal fever (MCF) on Swiss farms, where “exotic” water buffaloes were kept together with “native” animals, i.e. cattle, sheep, and goats. In the first farm with 56 water buffaloes, eight cases of MCF due to *ovine herpesvirus-2* (OvHV-2) were noted, whereas additional ten water buffaloes were subclinically infected with either OvHV-2 or *caprine herpesvirus-2* (CpHV-2). On the second farm, 13 water buffaloes were infected with CpHV-2 and two of those succumbed to MCF. In neither farm, any of the two viruses were detected in cattle, but the Macaviruses were present at high prevalence among their original host species, sheep and goats, respectively. On the third farm, sheep were kept well separated from water buffaloes and OvHV-2 was not transmitted to the buffaloes, despite of high prevalence of the virus among the sheep. Macavirus DNA was frequently detected in the nasal secretions of virus-positive animals and in one instance OvHV-2 was transmitted vertically to an unborn water buffalo calf. Thus, water buffaloes seem to be more susceptible than cattle to infection with either Macavirus; however, MCF did not develop as frequently. Therefore, water buffaloes seem to represent an interesting intermediate-type host for Macaviruses. Consequently, water buffaloes in their native, tropic environments may be vulnerable and endangered to viruses that originate from seemingly healthy, imported sheep and goats.

## Introduction

The water buffalo was introduced into Switzerland in 1996, as 5 farmers from a western-central region imported 14 pregnant water buffalo cows and one water buffalo bull from Romania. For many years, artificial insemination with semen imported from Italy was performed, in order to maintain a broad genetic basis among the water buffalo population [1]. Meanwhile, water buffaloes are held in 75 farms all over Switzerland (personal communication, Tierverkehrsdatenbank, 12-23-2011) and further breeding is possible without importing new animals.

The Mediterranean River Buffalo, antecessor of the Romanian and Italian buffalo breeds, originates from the Domestic Asian Water Buffalo (*Bubalus bubalis*) [2], [3]. Although water buffaloes seem to be susceptible to most diseases that affect cattle, showing some variety in sensitivity or resistance, they are generally known to be in good health and well adapted to the hot and humid climates of tropical countries with the corresponding pathogens. However, reactions to some diseases may vary depending on the region, environment and genetic basis of the buffalo breed [4]. Foot-and-mouth disease, Rinderpest, Infectious Bovine Rhinotracheitis, Bluetongue, Bovine Viral Diarrhea, Buffalo pox, Rabies, Ephemeral Fever and Malignant Catarrhal fever are some of the viral infections described in water buffaloes in Asia and Europe [5].

Malignant catarrhal fever (MCF) is an often-fatal lymphoproliferative disease of mainly ruminant species including domestic cattle, water buffalo, American bison, various species of cervids and other wild living ruminants, caused by closely related *Gammaherpesvirinae* of the genus *Macavirus* [6]. Two viruses are primarily responsible for the disease; the *ovine herpesvirus-2* (OvHV-2) as seen worldwide in sheep-associated-MCF (SA-MCF), with sheep as the main reservoir host [7]–[9], and the *alcelaphine herpesvirus-1* (AlHV-1), endemic in wildebeest, inducing wildebeest-associated MCF (WA-MCF) in Africa and zoological gardens [8]–[10]. In recent years, further MCF agents have been recognized; the MCF-causing virus in white-tailed deer [11], [12], cattle [13] and red brocket deer [14] with unknown reservoir host, although a potential connection with goats has been suggested [14], [15]; the ibex MCF virus with outbreaks of MCF in bongo antelopes [16], [17]; the AlHV-2-like virus introducing MCF in Barbary red deer [18]; and the *caprine herpesvirus-2* (CpHV-2) identified in healthy domestic goats [19]–[21], leading to clinical cases in different kinds of cervids [22]–[27] and probably in domestic cattle, banteng [13], [27] as well as in water buffaloes [28].

Clinical signs of MCF in water buffaloes are characterized by depression, anorexia, high fever, lymphadenopathy, conjunctivitis and corneal opacity, inflammation, ulceration and exudation of the upper digestive- and respiratory tract, diarrhea and neurological deficiencies leading to death [29]–[33].

In Switzerland, SA-MCF sporadically occurs in domestic cattle [34]; one case in a farmed sika deer has been mentioned [35]. OvHV-2 has been detected in pigs [36]. In water buffaloes, only one case of probable CpHV-2-associated MCF-like disease has been described [28].

Over the last few years the popularity of housing water buffaloes for milk and meat production has risen. Up to now approximately 1202 water buffaloes are kept in Switzerland, in smaller herds and herds of up to more than 50 animals (personal communication, Tierverkehrsdatenbank, 12-23-2011), distributed over all geographical regions. The economic loss in case of death is quite high. Preceding our survey, a Swiss farm reported increased fatalities following MCF-like symptoms in water buffaloes. For the current case report we therefore decided to investigate 2 Swiss water buffalo farms with history of disease and one farm with no known MCF-like case, by means of real-time PCR analysis, in order to detect OvHV-2 and/or CpHV-2 as probable triggers of disease and gain more insights concerning epidemiological issues.

## Materials and Methods

### 2.1. Ethics statement

This study was carried out in strict accordance with the Swiss regulations for animal experimentation. The protocol for this study was approved by the Cantonal Veterinary Office Zurich, ZH, Switzerland (Permit Number: 102/2012).

Moreover, the owners of the water buffaloes gave permission for their animals to be used in this study.

### 2.2. Animals

In the course of our study we investigated 3 Swiss farms housing water buffaloes (*Bubalus bubalis*) as well as additional ruminants. Switzerland is essentially free of *bovine herpesvirus-1* (BoHV-1) and keeps this state on the basis of serological surveys. Before granting import permits, it is mandatory to test susceptible cloven-hoofed animals, including water buffaloes, for antibodies against BoHV-1. Only seronegative animals will be issued with a permit for importation. Due to serological cross-reaction, antibodies against *bubaline herpesvirus-1* (BuHV-1) can be detected in the same test (Engels and Ackermann, personal observation). Moreover, for differential diagnostic reasons, several individuals with MCF (detected in our study) were tested with negative result for BoHV-1 antibodies. Additionally, a total of 31 buffaloes, including individuals from all 3 farms, were specifically tested for BoHV-1 and BuHV-1 shedding by real-time PCR (Ackermann and co-workers, unpublished data); all with negative result. Therefore, we assume that neither BoHV-1 nor BuHV-1 had any effect on the present study.

#### Farm 1

The first herd, located in the lowlands, consisted of 56 water buffaloes, including 53 cows and calves (not individually discriminated) and three adult bulls. During summertime the water buffaloes lived in a free-stall barn with periodical pasturing, in direct contact with 42 Holstein-Friesian cattle. Moreover, 60 Dorper sheep and 6 Boer goats were kept in a separate barn, approximately 30 m off the buffaloes' and cattle's barn. Some water buffaloes spent the summer on an external pasture belonging to a remote farm, where also sheep were kept (relevance for case No. 8). During autumn and wintertime the water buffaloes and cattle were housed indoors under the same roof together with the small ruminants. As Dorper sheep do not keep to a restricted lambing period, birth giving occurs year round. Milk lambs were allowed to roam freely among buffaloes and cattle, and occasionally fed from the same manger. The sheep and goats were removed from the premises in August 2011; 3–4 months after a first water buffalo came down with Malignant Catarrhal fever (MCF).

#### Farm 2

The second herd of 21 water buffaloes was held in the alpine regions of Switzerland. Throughout the year, including birth-giving times, the water buffaloes were housed in the same barn together with 4 cattle and 7 goats. They shared the summer pasture with the goats and additional cattle. Remaining feed of the goats as well as goat milk was occasionally fed to the water buffaloes. No sheep were kept on the farm, and no contact with external sheep is known.

#### Farm 3

The third herd of 43 water buffaloes was situated in the central region of Switzerland. Apart from the water buffaloes living in a free-stall barn with periodic access to the pasture, fluctuating numbers of 20 to 50 sheep of a Swiss breed (Schweizer Alpenschaf) were housed in a separate stable and separate pasture throughout the year. The sheep gave birth from January to March; no free roaming of offspring occurred on the premises. There was no known direct contact between sheep and water buffaloes. Neither cattle nor goats were kept on the same farm.

### 2.3. Sample collection

5–10 ml EDTA-treated blood was collected from 118 water buffaloes and 13 goats and from a selection of 17 cattle and 12 sheep. Additionally to the 160 blood samples nasal swabs were taken from 30 water buffaloes and six goats. From one water buffalo and 3 water buffalo fetuses only fresh organ samples could be collected. Two water buffalo calves from farm 1 were monitored beginning by time of birth by analyzing a monthly blood sample over a period of 8 months. The blood of 28 water buffaloes from farm 1 was re-tested 8 to 13 months after the first blood sampling.

### 2.4. Sample preparation and DNA extraction

Buffy-coat cells from each sample were gained through the addition of 40 ml lysis buffer (0.15 M NH_4_Cl, 10 mM CHKO_3_, 0.1 mM EDTA [pH 7.2]) to the EDTA-treated blood, followed by centrifugation for 10 min at 868× g and disposal of the supernatant. This step was repeated 1–3 times. The final pellet was re-suspended in 40 ml phosphate-buffered saline, and after a another centrifugation step and disposal of the supernatant, was stored at −20°C until further use. Dry nasal swabs were used directly for DNA extraction. Fresh organ samples were crushed before lysis and DNA extraction. DNA extraction was carried out using the QiAamp DNA Mini Kit (Qiagen, Hombrechtikon, Switzerland) according to the manufacturer's recommendations for each of the collected sample materials. The extracted DNA was used directly for PCR typing or stored at −20°C.

### 2.5. PCR

#### OvHV-2

The genomic DNA sequence of the ORF 63 tegument protein from OvHV-2 provided the basis for the design of the real-time PCR. The sequence described by Taus et al. [37] (GenBank accession no. DQ198083.1), as well as the sequence of Hart et al. [38] (GenBank accession no. AY839756.1), were considered. Primers and probe were designed using the Perkin-Elmer Primer Express software (version 1.0, Perkin-Elmer, Foster City, California). The following primers and probe were selected: forward primer 5′-GAG AAC AAG CGC TCC CTA CTG A-3′ (Life Technologies Europe BV, Zug, Switzerland), reverse primer 5′-CGT CAA GCA TCT TCA TCT CCA G-3′ (Life Technologies), probe 5′-FAM-AGT GAC TCA GAC GAT ACA GCA CGC GAC A-TAMRA-3′ (Microsynth, Balgach, Switzerland). Possible cross-reactions with other *Herpesvirales* were evaluated with the National Center for Biotechnology Information Basic Local Alignment Search Tool (NCBI BLAST, http://blast.ncbi.nlm.nih.gov/Blast.cgi). PCR reaction mixture and cycling conditions were carried out as described previously by Hüssy et al. [39] considering the above-mentioned modifications of primers and probe and run on an 7900HT Fast Real-Time PCR System (Life Technologies) with 9600 emulation ramping. 10 µl of diluted and undiluted samples were tested in duplicates.

#### CpHV-2

For the detection of CpHV-2 primers and probes described by Cunha et al. [40] were used. Real-time PCR was performed using the same conditions as for the OvHV-2 PCR. However only 5 µl of diluted or undiluted template DNA was added in duplicates and the ramping of the cycler was set to the standard rate.

#### 12S rDNA

The 12S rRNA gene was used as an internal control in order to confirm positive DNA extraction. The PCR was based on the finding of a consensus sequence between previously published [41] 12S rDNA sequences of various bovid taxa. 5′-GCG GTG CTT TAT AYC CTT CTA GAG-3′, 600 nM, served as forward primer, 5′-TTA GCA AGR ATT GGT GAG GTT TAT C-3′, 600 nM, (Microsynth, Balgach, Switzerland) as the reverse primer. The probe 5′-VIC-AGC CTG TTC TAT AAY CGA T-MGBNFQ-3′, 160 nM, (Life Technologies) was used. Samples from water buffaloes, cattle, sheep and goats were positively tested for the described house-keeping gene, confirming their sensitivity to the reaction. For real-time PCR the same conditions as mentioned above were used. 10 µl of the diluted sample in question applied in dual wells served as template.

## Results

### 3.1. Incidence of clinical MCF

A total of 4 severe and 4 relatively mild cases of MCF were noticed over a period of 17 months, from April 2011 to August 2012, among the water buffaloes of farm 1 (Table 1). In each single case OvHV-2 DNA was detected in the diseased animals. In farm 2, two cases of clinical MCF were reported, one in October 2008 and one in November 2010 but testing for OvHV-2 DNA remained negative. No cases of clinical MCF were reported from farm 3.

**Table 1:** Time scale of events and OvHV-2 real-time PCR results from water buffaloes with clinical MCF of farm 1.

Buffalo No. Clinical MCF	Apr-11	May-11	Jul-11	Aug-11*	Sep-11	Oct-11	Nov-11	Dec-11	Jan-12	May-12
severe										
**1**	OvHV-2+c, ^†^									
**2**	nd	OvHV-2+^c^	OvHV-2+c, ^†^							
**3**	nd	nd	OvHV-2+^c^	nd	OvHV-2 +c, ^†^					
**4**	nd	nd	nd	nd	nd	OvHV-2+c, ^†^				
mild										
**5**	nd	nd	OvHV-2+	nd	nd	nd	OvHV-2+c, ^†^			
**6**	nd	nd	Nd	nd	nd	nd	nd	OvHV-2+	OvHV-2+c, ^†^	
**7**	nd	nd	OvHV-2-	nd	nd	nd	nd	nd	OvHV-2+c, ^†^	
**8**	nd	nd	nd	nd	nd	nd	nd	OvHV-2-	nd	OvHV-2+c, ^†^

OvHV-2+  =  positive for OvHV-2;

OvHV-2-  =  negative for OvHV-2;

nd  =  not determined;

^c^ =  clinical signs of MCF;

† =  euthanasia or slaughter due to clinical MCF;

* =  sheep removed from farm.

Thus, despite the presence of small ruminants on all three farms, three different types of outcomes were noticed: (1) relatively frequent (almost monthly) cases of OvHV-2-associated MCF in water buffaloes of farm 1; (2) relatively low frequency of clinical MCF in water buffaloes without detection of OvHV-2 DNA on farm 2; (3) no evidence of clinical MCF on farm 3. Based on these observations, questions arose concerning the prevalence of OvHV-2 and other potential MCF agents among the small ruminants living on the same farms as well as about the possibility of subclinical circulation of MCF agents among the water buffaloes.

### 3.2. Search for OvHV-2 and CpHV-2 among cattle and small ruminants on the same farms

13 of 42 cattle on farm 1 were available for testing and all turned out to be negative for both viruses, OvHV-2 and CpHV-2. Since OvHV-2 is known to be highly prevalent in sheep, only 6 out of 60 sheep on farm 1 were tested. Indeed, 5 of those 6 sheep carried OvHV-2, suggesting a high prevalence of this MCF agent among the sheep of farm 1. In contrast, CpHV-2 DNA was not detected in the samples of the same sheep, suggesting no or a low prevalence of CpHV-2 among the sheep. Among the 6 goats on the same farm, one was identified as positive for CpHV-2 and another as positive for OvHV-2 DNA in blood and nasal swabs. No viral DNA was detected in a nasal swab sample from a third goat, which however tested positive for both OvHV-2 and CpHV-2 in the blood. Thus, at least two different MCF agents were present and apparently circulating in their primary hosts on farm 1.

In farm 2, the 4 cattle tested negatively for both OvHV-2 and CpHV-2, whereas CpHV-2 DNA was detected in the blood samples from 6 of the 7 co-housed goats. This was consistent with a high prevalence of CpHV-2 in its native host species. In contrast, OvHV-2 was not detected in the same goats.

In farm 3, again 6 individual sheep were randomly selected for testing against OvHV-2 and CpHV-2. All of them were shown to be infected with OvHV-2, but none of them provided evidence of CpHV-2 infection.

Thus, OvHV-2 circulated among sheep in farm 1 and farm 3, whereas CpHV-2 showed a high prevalence among the goats in farm 2 and a lesser prevalence among the goats in farm 1.

### 3.3. Prevalence of OvHV-2 and CpHV-2 among water buffaloes on the same farms

**Figure 1:**
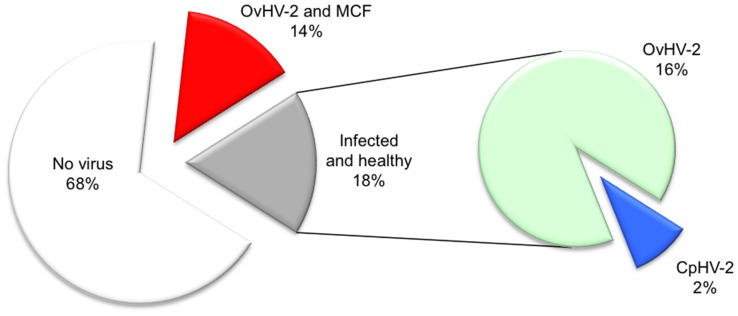
Proportion of Macavirus-affected water buffaloes on farm 1. Samples from water buffaloes (n = 56) were analyzed by real-time PCR for the detection of OvHV-2 DNA and CpHV-2 DNA, respectively. The figure plots the percentage of healthy animals (“No virus detected”: white; “infected and healthy”: grey) versus the proportion of animals with MCF due to OvHV2 infection (red). In the secondary pie, the proportion of infected but healthy animals is further subdivided into animals with OvHV2 (green) and CpHV2 (blue), respectively.

Fig. 1 shows that both MCF agents present on farm 1, OvHV-2 and CpHV-2, had taken an opportunity to infect water buffaloes. However, CpHV-2 caused only one subclinical infection among the 56 water buffaloes, whereas OvHV-2 was associated with 8 cases of clinical MCF and 9 incidences of subclinical infections on the same farm. Thus, OvHV-2 infections among water buffaloes in this farm amounted to a prevalence of about 30%, but only 50% of the infected individuals succumbed to MCF. These observations indicate that, with equal frequency, OvHV-2 infections of water buffaloes may either take a subclinical or a manifest course with respect to MCF. Interestingly, one of the eight water buffaloes with clinical MCF (case No. 8, Table 1) tested OvHV-2- negative in December 2011. However, five months later and a total of 9 months after the OvHV-2-positive sheep had been removed from the farm, the same animal fell ill with OvHV-2-associated MCF. In this case, the source of OvHV-2 remains obscure.

Analysis of nasal swab samples from farm 1 revealed that in 3 out of 3 water buffaloes with clinical MCF and in 5 of 10 subclinically OvHV-2-infected water buffaloes the agent could simultaneously be detected in white blood cells and in nasal secretions. In contrast, excretion of CpHV-2 could not be detected in the nasal sample of the one CpHV-2-positive water buffalo.

Organs of 2 fetuses from seriously diseased, OvHV-2-positive water buffaloes of farm 1 were also analyzed; one fetus was proven to be positive for OvHV-2 in the spleen. Thus, vertical transmission of OvHV-2 in water buffaloes seemed to be possible. In an organ pool of fresh brain, kidney, heart and intestine of the second fetus, no virus was detected. Unfortunately no spleen sample was available from this second fetus. The only CpHV-2 positive water buffalo of farm 1 had an abortion without showing further clinical symptoms. Fresh fetal brain and lymph node samples were tested negative for both viruses; no obvious reason of prenatal death could be found. Thus, vertical transmission of CpHV-2 among water buffaloes could not be substantiated.

**Figure 2:**
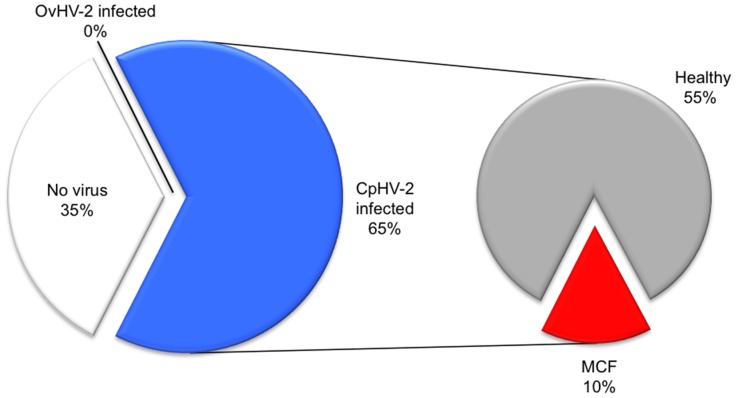
Proportion of Macavirus-affected water buffaloes on farm 2. Samples from water buffaloes (n = 20) were analyzed by real-time PCR for the detection of OvHV-2 DNA and CpHV-2 DNA, respectively. The figure plots the percentage of virus-free (“No virus detected”: white) and virus-infected animals (“OvHV2 infected”: black; “CpHV2 infected”: blue). In the secondary pie, the proportion of CpHV2-infected animals is further subdivided into animals with MCF (red) and healthy individuals (grey).

Fig. 2 shows that water buffaloes may be very susceptible to CpHV-2. With 13 positive individuals among 20 animals tested on farm 2, the prevalence amounted to 65%. However, only two out of those 13 animals did succumb to an MCF-like disease. In the absence of OvHV-2, these observations strongly suggest that CpHV-2 was the most likely agent for the disease. However, the subclinical course with CpHV-2 was much more frequent than with OvHV-2 in farm 1.

Interestingly, CpHV-2 DNA was only detected in the nasal swab from one out of 11 CpHV-2-positive animals. Therefore, independent circulation of CpHV-2 among water buffaloes was not strongly supported by our data.

Remarkably, despite of its apparently high prevalence among the sheep in farm 3, transmission of OvHV-2 from the reservoir host to water buffaloes was not detected and clinical cases did not occur. Thus, strict separation of sheep and water buffaloes on such farms may be effective to prevent MCF.

## Discussion

Considering the ongoing global spread of “exotic” farm animals from their native to novel environments, it is imperative to keep in mind that they all carry their own sets of viruses, which may be well adapted to their original hosts, but may bear unknown risks for the animals, into whose ecological niches they are introduced. In contrast, “exotic” farm animals may be especially sensitive for viruses circulating in foreign countries and various MCF-causing agents seem to be an interesting topic in this context.

For the present communication, we started off with clinical cases of malignant catarrhal fever (MCF) in water buffaloes (considered “exotic”) from two Swiss farms and, consecutively, analyzed the genoprevalence of two different MCF agents, OvHV-2 and CpHV-2, in various susceptible ruminants on the same farms. A third farm, from which no cases of MCF had been reported, served as comparison. The following insights were gained:

1. Our report confirms earlier work mentioning that water buffaloes were more susceptible to OvHV-2 infection than cattle [8], [31], [33], [42]. As expected, sheep and goats, the reservoir hosts of OvHV-2 and CpHV-2, respectively, were shown to be Macavirus-carriers in both, farm 1 and farm 2. Yet, despite similar exposure, cases of MCF as well as subclinical genoprevalence of the MCF agents were noted only in the water buffaloes but not in cattle. It is well known that susceptibility to infection by Macaviruses may depend on animal species. Certain species of deer (i.e. Père David's deer [43]), Bali cattle [42], [44] as well as American bison [45]-[49] are considered highly susceptible to OvHV-2.
2. Despite this increased susceptibility, the mere presence of Macavirus-carriers on the farms did not appear to be sufficient for the transmission of the two MCF agents from the reservoir hosts to the water buffaloes as indicator hosts. For example, transmission of CpHV-2 from the infected goats to water buffaloes was only observed in one case on farm 1, whereas the genoprevalence of CpHV-2 amounted to 65% among the water buffaloes on farm 2. The true reason for this difference could not be determined under the present conditions. However, possible explanations for the high CpHV-2 transmission rate in farm 2 may be the joint keeping of goats and water buffaloes on pastures and in the stable throughout the entire year. Moreover, the water buffaloes were occasionally fed with remaining feed and milk of the goats. Alternatively, one may consider independent circulation of CpHV-2 among water buffaloes. However, the virus was detected only in one out of 11 nasal swabs taken from CpHV-2-positive water buffaloes of farm 2, which did not heavily support this possibility.
  OvHV-2 was highly prevalent in sheep on farm 1 as well as on farm 3. Whereas, on farm 3, the virus was not transmitted to the water buffaloes, it caused subclinical infection and mild to severe MCF outbreaks among the water buffaloes on farm 1. The obvious explanation for this may be attributed to the different types of management on the two farms: whereas the water buffaloes and the sheep had been co-stabled at winter time in farm 1, they were housed and grazed separately in farm 3, which may be considered a successful preventive measure. Although long distance spread of OvHV-2 from sheep to susceptible animals, such as bisons, has been reported by others [49], close contact among sheep and other susceptible species, especially keeping them under the same roof, is considered as a more efficient means to transmit OvHV-2 [50], [51]. Also, the temporary free ranging of hand-raised lambs in the water buffalo stable of farm 1 seems to favor OvHV-2 transmission. The role of the OvHV-2 infected goats of farm 1 is not clear. OvHV-2 DNA in blood samples and nasal swabs of goats housed together with sheep has been detected previously [15], [19], [21], [27], [44], [52]. In cattle and bison without clinical signs of MCF, DNA of OvHV-2 has been extracted from blood, milk, nasal secretions and conjunctiva [13], [46]–[48], [50], [51], [53], [54]
  It is unclear whether the OvHV-2 and CpHV-2-positive results from healthy water buffaloes in farms 1 and 2 were merely due to ongoing prolonged incubation periods prior to an MCF outbreak or else were attributable to water buffaloes as potential reservoir hosts.
  Although the detection of OvHV-2 DNA in nasal secretions does not provide direct proof for virus excretion, the relatively high frequency of detection of OvHV-2 DNA in nasal swabs of infected water buffaloes on farm 1, might support the notion that OvHV-2 may circulate by horizontal transmission among water buffaloes in a sheep-independent manner. However, re-testing of blood samples of originally virus-free water buffaloes in farm 1, at eight to 13 months after removal of the sheep from the premises, did not reveal any newly infected carriers of OvHV-2. Similarly, two water buffalo calves, born after the removal of the sheep, were monitored at monthly intervals for OvHV-2. Neither calf turned OvHV-2-positive until the monitoring was ceased seven months later.
  The source of infection for water buffalo No. 8 from farm 1 remains unclear. Since the water buffalo spent the summer months of 2011 on an external farm, which also housed sheep, an infection on those premises must be considered. However, the summering farm housed various water buffaloes and to our knowledge no further case of MCF had been registered. Indirect transmission, as well as direct horizontal transmission from a subclinically infected water buffalo as reservoir host, cannot be ruled out. A further possible explanation is that the virus load of the sample taken in December 2011 was under the detection limit of real-time PCR analysis; temporary variation of OvHV-2 DNA detection in peripheral blood lymphocytes of susceptible hosts has previously been demonstrated in cattle [51], [53]–[55] and bison [56], [57].
  Interestingly, OvHV-2-DNA was detected in the spleen of a fetus from a deceased, OvHV-2-positive dam of farm 1, which confirms potential trans-placental transmission of OvHV-2 among water buffaloes. To our knowledge, this might represent the first report on vertical Macavirus transfer among MCF indicator hosts. Yet, as no spleen samples were available in the cases of the other two examined fetuses of a further OvHV-2 and of a CpHV-2 positive dam, no definitive conclusion can been drawn concerning the frequency of such a transmission. Vertical transmission seems to play an important role in wildebeest, the reservoir host of AlHV-1 [10], [58], [59]. In contrast, in sheep, vertical transmission of OvHV-2 may occur only rarely [60], whereas trans-placental transmission from CpHV-2 positive goats to their kids seems not to play a major role [21]. It will be important to address these issues more deeply, especially with regard to water buffaloes.
3. Despite the considerations mentioned above, the water buffaloes seemed to be generally more susceptible to CpHV-2 (two-thirds of exposed animals positive) than to OvHV-2 (one-third positive). However, with 50% of infected water buffaloes of farm 1 succumbing to MCF due to OvHV-2, compared to only 15% of the CpHV-2-positive animals developing MCF on farm 2, the penetration rate of MCF appeared to be higher with OvHV-2 than with CpHV-2. Compared to cattle, the best studied indicator host, water buffaloes seem to be more susceptible to Macavirus infections but less prone to succumb to MCF. However, in comparison to typical reservoir hosts, water buffaloes seem to be less susceptible to Macavirus infections but more prone to subsequently succumb to MCF. Thus, they may represent an interesting intermediate-type host for the Macaviruses.

Therefore, it may be important to extend the research on MCF not only to relatively poorly characterized viruses, such as CpHV-2, but also to thus far neglected indicator host species, such as water buffaloes. Indeed, MCF is of great concern among different species of buffaloes, particularly in Asia, but also in Brazil and Italy [29], [30], [42]; clinical MCF in free-ranging African buffaloes is considered an emerging disease [61]. In association with shared housing of water buffaloes and sheep and the frequency of MCF, OvHV-2 seems to be of high relevance [29]–[31]. However, our investigation confirms that water buffaloes can also succumb to MCF due to CpHV-2 [28]. Hence, shared housing of water buffaloes and goats has to be considered as a risk factor for MCF as well.

In the present case report, the question whether or not Macaviruses actively circulate among water buffaloes could not be unanimously solved. The notion that Macavirus DNAs were detected in nasal secretions of virus-positive animals and in the spleen of one fetus seems to argue in favor of active virus transmission among water buffaloes. However, after the removal of the original reservoir hosts from one farm, newly infected individuals, with just one exception (case No. 8, Table 1), could not be identified, which rather speaks against efficient intra-species transmission.

In conclusion, two different Macaviruses, OvHV-2 and CpHV-2, behaved differently in the “exotic” host, the water buffalo, than in “native” host species, such as cattle, sheep, and goats. On the one hand, the water buffaloes seemed to be more susceptible than cattle to infection with either virus; on the other hand, the case fatality rate among water buffaloes was lower than the perceived lethality among cattle [34]. However, recent reports from countries with a high concentration of farms, where OvHV-2 reservoir species are kept together with MCF-indicator species, suggested an increasing incidence of non-lethal outcomes of MCF, which also involved either subclinical cases among cattle, and more chronic, cutaneous forms of the disease [50], [62]–[64]. These observations may signify that either OvHV-2 has a capacity to adapt to new hosts upon serial passaging or else that the cattle in those countries are selected to become resistant to MCF. Both possibilities would be worthwhile to consider further.

Finally, it may be valuable to also look into the water buffalo's immune responses against the different types of Macaviruses in order to better understand the reasons for the relatively high number of subclinical cases.

## References

[1] Rüeger R, Spring P, Linder HJ (2010) Wasserbüffel in Schangnau. ValiDist Post Vertiefungsarbeit. Wasserbüffel in Schangnau website. Available: http://www.wasserbueffel-schangnau.ch. Accessed 2013 Nov 19.

[2] Borghese A, Mazzi M (2005) Buffalo Population and Strategies in the World. In: Borghese A, editor. Buffalo Production and Research. Rome: Food and Agriculture Organization of the United Nations. pp. 1–39.

[3] Moioli B, Borghese A (2005) Buffalo Breeds and Management Systems. In: Borghese A, editor. Buffalo production and research. Rome: Food and Agriculture Organization of the United Nations. pp. 51–76.

[4] National Research Council (1981) The Water Buffalo: New Prospects for an Underutilized Animal. Washington: Board on Science and Technology for International Development. 111 p.

[5] Fagiolo A, Roncoroni C, Lai O, Borghese A (2005) Buffalo Pathologies. In: Borghese A, editor. Buffalo production and research. Rome: Food and Agriculture Organization of the United Nations. pp. 249–289.

[6] Davison AJ, Eberle R, Ehlers B, Hayward GS, McGeoch DJ, et al (2008) The order Herpesvirales. Arch Virol 154: 171–177. 19066710 10.1007/s00705-008-0278-4 PMC3552636

[7] Baxter SI, Wiyono A, Pow I, Reid HW (1997) Identification of ovine herpesvirus-2 infection in sheep. Arch Virol 142: 823–831. 9170507 10.1007/s007050050121

[8] Reid HW, Buxton D (1989) Malignant catarrhal fever and the gammaherpesvirinae of bovidae. Developments in Veterinary Virology 9: 116–162.

[9] Plowright W (1990) Malignant catarrhal fever virus. In: Dinter Z, Morein B, editors. Virus infections of ruminants. New York: Elsevier Science Publishers B.V. pp. 123–150.

[10] Plowright W, Ferris RD, Scott GR (1960) Blue Wildebeest and the Aetiological Agent of Bovine Malignant Catarrhal Fever. Nature 188: 1167–1169. 13736396 10.1038/1881167a0

[11] Kleiboeker SB, Miller MA, Schommer SK, Ramos-Vara JA, Boucher M, et al (2002) Detection and multigenic characterization of a herpesvirus associated with malignant catarrhal fever in white-tailed deer (Odocoileus virginianus) from Missouri. J Clin Microbiol 40: 1311–1318. 11923350 10.1128/JCM.40.4.1311-1318.2002 PMC140372

[12] Li H, Dyer N, Keller J, Crawford TB (2000) Newly recognized herpesvirus causing malignant catarrhal fever in white-tailed deer (Odocoileus virginianus). J Clin Microbiol 38: 1313–1318. 10747100 10.1128/jcm.38.4.1313-1318.2000 PMC86438

[13] Cissell RL (2010) Malignant Catarrhal Fever Viruses in Tennessee Ruminants. The University of Tennessee Knoxville website. Available: http://trace.tennessee.edu/utk_graddiss/786/. Accessed: 19 Nov 2013.

[14] Li H, Cunha CW, Abbitt B, deMaar TW, Lenz SD, et al (2013) Goats are a potential reservoir for the herpesvirus (MCFV-WTD), causing malignant catarrhal fever in deer. J Zoo Wildl Med 44: 484–486. 23805572 10.1638/2012-0087R.1

[15] Matzat T (2012) Beitrag zum Bösartigen Katarrhalfieber bei Wiederkäuern in zoologischen Gärten. Quality Content of Saxony website. Available: http://www.qucosa.de/recherche/frontdoor/?tx_slubopus4frontend[id] = 8349. Accessed: 19 Nove 2013.

[16] Okeson DM, Garner MM, Taus NS, Li H, Coke RL (2007) Ibex-associated malignant catarrhal fever in a bongo antelope (Tragelaphus euryceros). J Zoo Wildl Med 38: 460–464. 17939356 10.1638/06-046.1

[17] Gasper D, Barr B, Li H, Taus N, Peterson R, et al (2012) Ibex-associated malignant catarrhal fever-like disease in a group of bongo antelope (Tragelaphus eurycerus). Vet Pathol 49: 492–497. 22194357 10.1177/0300985811429306

[18] Klieforth R, Maalouf G, Stalis I, Terio K, Janssen D, et al (2002) Malignant catarrhal fever-like disease in Barbary red deer (Cervus elaphus barbarus) naturally infected with a virus resembling alcelaphine herpesvirus 2. J Clin Microbiol 40: 3381–3390. 12202582 10.1128/JCM.40.9.3381-3390.2002 PMC130662

[19] Li H, Keller J, Knowles DP, Crawford TB (2001) Recognition of another member of the malignant catarrhal fever virus group: an endemic gammaherpesvirus in domestic goats. J Gen Virol 82: 227–232. 11125175 10.1099/0022-1317-82-1-227

[20] Chmielewicz B, Goltz M, Ehlers B (2001) Detection and multigenic characterization of a novel gammaherpesvirus in goats. Virus Res 75: 87–94. 11311431 10.1016/s0168-1702(00)00252-5

[21] Li H, Keller J, Knowles DP, Taus NS, Oaks JL, et al (2005) Transmission of caprine herpesvirus 2 in domestic goats. Vet Microbiol 107: 23–29. 15795075 10.1016/j.vetmic.2005.01.014

[22] Foyle KL, Fuller HE, Higgins RJ, Russell GC, Willoughby K, et al (2009) Malignant catarrhal fever in sika deer (Cervus nippon) in the UK. Vet Rec 165: 445–447. 19820260 10.1136/vr.165.15.445

[23] Keel MK, Gage PJ, Noon TH, Bradley GA, Collins JK (2003) Caprine Herpesvirus-2 in Association with Naturally Occurring Malignant Catarrhal Fever in Captive Sika Deer (Cervus Nippon). J Vet Diagn Invest 15: 179–183. 12661731 10.1177/104063870301500215

[24] Crawford TB, Li H, Rosenburg SR, Norhausen RW, Garner MM (2002) Mural folliculitis and alopecia caused by infection with goat-associated malignant catarrhal fever virus in two sika deer. J Am Vet Med Assoc 221: 843–7–801. 12322924 10.2460/javma.2002.221.843

[25] Li H, Wunschmann A, Keller J, Hall DG, Crawford TB (2003) Caprine Herpesvirus-2-Associated Malignant Catarrhal Fever in White-Tailed Deer (Odocoileus Virginianus). J Vet Diagn Invest 15: 46–49. 12580295 10.1177/104063870301500110

[26] Vikøren T, Li H, Lillehaug A, Jonassen CM, Böckerman I, et al (2006) Malignant catarrhal fever in free-ranging cervids associated with OvHV-2 and CpHV-2 DNA. J Wildl Dis 42: 797–807. 17255446 10.7589/0090-3558-42.4.797

[27] Förster C (2011) Gammaherpesviren bei kleinen Wiederkäuern, Zoo- und Wildtieren. Justus-Liebig-Universität Giessen website. Available: http://geb.uni-giessen.de/geb/volltexte/2011/8212/. Accessed: 19 Nov 2013.

[28] Dettwiler M, Stahel A, Krüger S, Gerspach C, Braun U, et al. (2011) A possible case of caprine-associated malignant catarrhal fever in a domestic water buffalo (Bubalus bubalis) in Switzerland. BMC Veterinary Res 7: : 78. Available: http://www.biomedcentral.com/1746-6148/7/78. Accessed: 19 Nov 2013. 10.1186/1746-6148-7-78 PMC3259070 22132808

[29] Martucciello A, Marianelli C, Capuano M, Astarita S, Alfano D, et al (2006) An outbreak of malignant catarrhal fever in Mediterranean water buffalo (Bubalus bubalis). Large Animal Review 12: 21–24.

[30] Costa ÉA, Bastianetto E, Vasconcelos AC, Bomfim MRQ, Fonseca FGD, et al (2009) An outbreak of malignant catarrhal fever in Murrah buffaloes in Minas Gerais, Brazil. Pesq Vet Bras 29: 395–400.

[31] Teankam K, Tantilertcharoen R, Boonserm T (2006) Malignant catarrhal fever in swamp buffaloes (Bubalus bubalis): A retrospective pathological study of outbreaks in Thailand. Thai J Vet Med 36: 19–30.

[32] Hill FI, Arthur DG, Thompson J (1993) Malignant catarrhal fever in a swamp buffalo (Bubalus bubalis) calf in New Zealand. New Zeal Vet J 41: 35–38. 10.1080/00480169.1993.35732 16031692

[33] Hoffmann D, Soeripto S, Sobironingsih S, Campbell RSF, Clarke BC (1984) The clinico-pathology of a malignant catarrhal fever syndrome in the Indonesian swamp buffalo (Bubalus bubalis). Aust Vet J 61: 108–112. 6540080 10.1111/j.1751-0813.1984.tb07201.x

[34] Müller-Doblies UU, Egli J, Li H, Braun U, Ackermann M (2001) Malignant catarrhal fever in Switzerland. 1. Epidemiology. Schweiz Arch Tierheilkd 143: 173–183. 11344942

[35] Sieber V, Robert N, Schybli M, Sager H, Miserez R, et al. (2010) Causes of Mortality and Diseases in Farmed Deer in Switzerland. Vet Med Int 2010: : 684924. Available: http://www.hindawi.com/journals/vmi/2010/684924/. Accessed: 19 Nov 2013. 10.4061/2010/684924 PMC2913629 20706668

[36] Albini S, Zimmermann W, Neff F, Ehlers B, Häni H, et al (2003) Identification and Quantification of Ovine Gammaherpesvirus 2 DNA in Fresh and Stored Tissues of Pigs with Symptoms of Porcine Malignant Catarrhal Fever. J Clin Microbiol 41: 900–904. 12574312 10.1128/JCM.41.2.900-904.2003 PMC149657

[37] Taus NS, Herndon DR, Traul DL, Stewart JP, Ackermann M, et al (2007) Comparison of ovine herpesvirus 2 genomes isolated from domestic sheep (Ovis aries) and a clinically affected cow (Bos bovis). J Gen Virol 88: 40–45. 17170434 10.1099/vir.0.82285-0

[38] Hart J, Ackermann M, Jayawardane G, Russell G, Haig DM, et al (2007) Complete sequence and analysis of the ovine herpesvirus 2 genome. J Gen Virol 88: 28–39. 17170433 10.1099/vir.0.82284-0

[39] Hüssy D, Stäuber N, Leutenegger CM, Rieder S, Ackermann M (2001) Quantitative fluorogenic PCR assay for measuring ovine herpesvirus 2 replication in sheep. Clin Diagn Lab Immunol 8: 123–128. 11139205 10.1128/CDLI.8.1.123-128.2001 PMC96020

[40] Cunha CW, Otto L, Taus NS, Knowles DP, Li H (2009) Development of a Multiplex Real-Time PCR for Detection and Differentiation of Malignant Catarrhal Fever Viruses in Clinical Samples. J Clin Microbiol 47: 2586–2589. 19494077 10.1128/JCM.00997-09 PMC2725674

[41] Gatesy J (1997) A Cladistic Analysis of Mitochondrial Ribosomal DNA from the Bovidae. Mol Phylogenet Evol 7: 303–319. 9187090 10.1006/mpev.1997.0402

[42] Daniels PW, Sudurisman WA, Wiyono A, Ronohardjo P (1988) Epidemiological aspects of malignant catarrhal fever in Indonesia. In: Daniels PW, Sudurisman WA, Ronohardjo P, editors. Malignant Catarrhal Fever in Asian Livestock. Canberra: Food and Agriculture Organization of the United Nations. pp. 20–31.

[43] Reid H, Buxton D, McKelvey W, Milne J, Appleyard W (1987) Malignant catarrhal fever in Pere David's deer. Vet Rec 121: 276–277. 3672838 10.1136/vr.121.12.276

[44] Wiyono A, Baxter SIF, Saepulloh M, Damayanti R, Daniels P, et al (1994) PCR detection of ovine herpesvirus-2 DNA in Indonesian ruminants – normal sheep and clinical cases of malignant catarrhal fever. Vet Microbiol 42: 45–52. 7839584 10.1016/0378-1135(94)90076-0

[45] Schultheiss PC, Collins JK, Austgen LE, DeMartini JC (1998) Malignant Catarrhal Fever in Bison, Acute and Chronic Cases. J Vet Diagn Invest 10: 255–262. 9683074 10.1177/104063879801000305

[46] Collins JK, Bruns C, Vermedahl TL, Schiebel AL, Jessen MT, et al (2000) Malignant Catarrhal Fever: Polymerase Chain Reaction Survey for Ovine Herpesvirus 2 and Other Persistent Herpesvirus and Retrovirus Infections of Dairy Cattle and Bison. J Vet Diagn Invest 12: 406–411. 11021426 10.1177/104063870001200503

[47] Li H, Taus NS, Jones C, Murphy B, Evermann JF, et al (2006) A devastating outbreak of malignant catarrhal fever in a bison feedlot. J Vet Diagn Invest 18: 119–123. 16566270 10.1177/104063870601800120

[48] O'Toole D, Li H, Sourk C, Montgomery DL, Crawford TB (2002) Malignant Catarrhal Fever in a Bison (Bison Bison) Feedlot, 1993–2000. J Vet Diagn Invest 14: 183–193. 12033673 10.1177/104063870201400301

[49] Li H, Karney G, O'Toole D, Crawford TB (2008) Long distance spread of malignant catarrhal fever virus from feedlot lambs to ranch bison. Can Vet J 49: 183–185. 18309750 PMC2216446

[50] Løken T, Bosman A-M, van Vuuren M (2009) Infection with Ovine herpesvirus 2 in Norwegian herds with a history of previous outbreaks of malignant catarrhal fever. J Vet Diagn Invest 21: 257–261. 19286510 10.1177/104063870902100216

[51] Rohlederer BS (2010) Untersuchungen zur Prävalenz von Infektionen mit dem ovinen Herpesvirus-2 (OvHV-2) in 20 Mischbetrieben mit Rindern und Schafen in Bayern. Ludwig-Maximilians-Universität München website. Available: http://edoc.ub.uni-muenchen.de/11904/. Accessed: 19 Nov 2013.

[52] Wiyono A (1999) The detection of ovine herpesvirus-2 in reservoir host of malignant catarrhal fever in Indonesia by means of polymerase chain reaction. Jurnal Ilmu Ternak dan Veteriner 4: 121–127.

[53] Powers JG, VanMetre DC, Collins JK, Dinsmore RP, Carman J, et al (2005) Evaluation of ovine herpesvirus type 2 infections, as detected by competitive inhibition ELISA and polymerase chain reaction assay, in dairy cattle without clinical signs of malignant catarrhal fever. J Am Vet Med Assoc 227: 606–611. 16117071 10.2460/javma.2005.227.606

[54] Strohbücker S (2005) Vorkommen und klinische Bedeutung von Infektionen mit dem Ovinen Herpesvirus 2 (OHV-2) bei Rindern und Schafen. Justus-Liebig-Universität Giessen website. Available: http://geb.uni-giessen.de/geb/volltexte/2006/2678/. Accessed: 19 Nov 2013.

[55] Taus NS, Oaks JL, Gailbreath K, Traul DL, O'Toole D, et al (2006) Experimental aerosol infection of cattle (Bos taurus) with ovine herpesvirus 2 using nasal secretions from infected sheep. Vet Microbiol 116: 29–36. 16621344 10.1016/j.vetmic.2006.03.005

[56] O'Toole D, Taus NS, Montgomery DL, Oaks JL, Crawford TB, et al (2007) Intra-nasal inoculation of American bison (Bison bison) with ovine herpesvirus-2 (OvHV-2) reliably reproduces malignant catarrhal fever. Vet Pathol 44: 655–662. 17846237 10.1354/vp.44-5-655

[57] Gailbreath KL, O'Toole D, Taus NS, Knowles DP, Oaks JL, et al (2010) Experimental nebulization of American bison (Bison bison) with low doses of ovine herpesvirus 2 from sheep nasal secretions. Vet Microbiol 143: 389–393. 20018461 10.1016/j.vetmic.2009.11.026

[58] Castro AE, Ramsay EC, Dotson JF, Schramke ML, Kocan AA, et al (1984) Characteristics of the herpesvirus of malignant catarrhal fever isolated from captive wildebeest calves. Am J Vet Res 45: 409–415. 6324620

[59] Barnard B (1990) Epizootology of wildebeest-derived malignant catarrhal fever: possible transmission among cows and their calves in the north-western Transvaal. Onderstepoort J Vet Res 57: 201–204. 2234868

[60] Li H, Taus NS, Lewis GS, Kim O, Traul DL, et al (2004) Shedding of ovine herpesvirus 2 in sheep nasal secretions: the predominant mode for transmission. J Clin Microbiol 42: 5558–5564. 15583281 10.1128/JCM.42.12.5558-5564.2004 PMC535255

[61] Pfitzer S, Last R, Espie I, van Vuuren M (2013) Malignant Catarrhal Fever: An Emerging Disease in the African Buffalo (Syncerus caffer). Transbound Emerg Dis In press. 10.1111/tbed.12131 23957274

[62] Munday JS, French AF, Smith A, Wang J, Squires RA (2008) Probable malignant catarrhal fever presented as transient generalised crusting dermatitis in a cow. New Zeal Vet J 56: 89–93. 10.1080/00480169.2008.36815 18408797

[63] David D, Dagoni I, Garazi S, Perl S, Brenner J (2005) Two cases of the cutaneous form of sheep-associated malignant catarrhal fever in cattle. Vet Rec 156: 118–120. 15704556 10.1136/vr.156.4.118

[64] Yeşilbağ K (2007) Seroprevalence of malignant catarrhal fever-related gammaherpesviruses in domestic ruminants in Turkey. Trop Anim Health Prod 39: 363–368. 17944306 10.1007/s11250-007-9024-2

